# Zn^2+^ Differentially Influences the Neutralisation of Heparins by HRG, Fibrinogen, and Fibronectin

**DOI:** 10.3390/ijms242316667

**Published:** 2023-11-23

**Authors:** Amélie I. S. Sobczak, Ramzi A. Ajjan, Alan J. Stewart

**Affiliations:** 1 School of Medicine, University of St Andrews, St. Andrews KY16 9TF, UK; amelie.sobczak26@gmail.com; 2 Leeds Institute of Cardiovascular and Metabolic Medicine, University of Leeds, Leeds LS2 3AA, UK; r.ajjan@leeds.ac.uk

**Keywords:** coagulation factors, factor Xa, fibrinogen/fibrin, thrombin, zinc

## Abstract

For coagulation to be initiated, anticoagulant glycosaminoglycans (GAGs) such as heparins need to be neutralised to allow fibrin clot formation. Platelet activation triggers the release of several proteins that bind GAGs, including histidine-rich glycoprotein (HRG), fibrinogen, and fibronectin. Zn^2+^ ions are also released and have been shown to enhance the binding of HRG to heparins of a high molecular weight (HMWH) but not to those of low molecular weight (LMWH). The effect of Zn^2+^ on fibrinogen and fibronectin binding to GAGs is unknown. Here, chromogenic assays were used to measure the anti-factor Xa and anti-thrombin activities of heparins of different molecular weights and to assess the effects of HRG, fibrinogen, fibronectin, and Zn^2+^. Surface plasmon resonance was also used to examine the influence of Zn^2+^ on the binding of fibrinogen to heparins of different molecular weights. Zn^2+^ had no effect on the neutralisation of anti-factor Xa (FXa) or anti-thrombin activities of heparin by fibronectin, whereas it enhanced the neutralisation of unfractionated heparin (UFH) and HMWH by both fibrinogen and HRG. Zn^2+^ also increased neutralisation of the anti-FXa activity of LMWH by fibrinogen but not HRG. SPR showed that Zn^2+^ increased fibrinogen binding to both UFH and LMWH in a concentration-dependent manner. The presented results reveal that an increase in Zn^2+^ concentration has differential effects upon anticoagulant GAG neutralisation by HRG and fibrinogen, with implications for modulating anti-coagulant activity in plasma.

## 1. Introduction

Neutralisation of anticoagulant glycosaminoglycans (GAGs) occurs prior to coagulation such that thrombin, factor Xa (FXa), and the other components of the coagulation cascade can be activated to facilitate fibrinogenesis [1,2]. Platelets, once active, aid this process through the release of various proteins that include histidine-rich glycoprotein (HRG), fibrinogen, and fibronectin. These proteins bind to anticoagulant GAGs to prevent their interaction with antithrombin (AT) [2]. Such binding and neutralisation events can depend upon the chain length of the GAGs. This is important, as low-molecular-weight heparins (LMWHs; ≤10,000 Da) are widely used as anti-thrombotic drugs [1,2,3].

In addition to releasing GAG-neutralising proteins, activated platelets also release metal ions that modulate the coagulation cascade. These include Zn^2+^, which both enhances and suppresses coagulation (as has been reviewed by others [4,5]) and acts to enhance GAG–protein interactions [3]. In humans, approximately 75% of the total 15–20 µM plasma zinc is bound to human serum albumin (HSA) [6], constituting >99% of the labile Zn^2+^ pool [7]. Most of the remaining (labile) Zn^2+^ is bound to small molecules, and a study in rats suggested that around ~1–3 nM Zn^2+^ may be “free” under resting conditions [8]. However, the available concentration of zinc at the surface of a platelet immediately following release is likely to be much higher.

In the absence of Zn^2+^, HRG binds unfractionated heparin (UFH) with a K_d_ of 32.9 nM, forming a 1:1 complex; this binding is unaffected by heparin chain length [9]. On the other hand, in the presence of 1 µM Zn^2+^, HRG binds high-molecular-weight heparin molecules (HMWH; ≥10,000 Da) with a K_d_ of 5.1 nM and forms a 1:2 complex [9,10]. Interestingly, the presence of Zn^2+^ does not appear to affect the binding of LMWH to HRG [9]. Another GAG-binding protein is fibrinogen, which binds UFH with K_d_ of 228 nM [11], which decreases to 60 nM in the presence of 12.5 µM Zn^2+^ [12]. In the cases of HRG and fibrinogen, it is not known if the Zn^2+^-mediated enhancement of GAG binding affinity results in an increase in GAG neutralisation, which may have clinical implications. The influence of GAG chain length on binding to fibrinogen and whether there are differential effects on affinity (dependent on chain length) mediated by Zn^2+^ are also unknown. Fibronectin binds a 6-saccharide heparin (1950 kDa) with a K_d_ of 18 µM and to 18- to 20-saccharide heparins (6000 Da) with a K_d_ of 0.9 µM [13]. The nucleation of fibronectin fibril assembly requires heparin binding [14]. Interestingly, a heparin-binding fragment of the protein was recently demonstrated to aid the integration of titanium-based dental implants [15]. However, the binding affinities of fibronectin to longer-chain heparins are not yet known. Given that fibronectin can also bind Zn^2+^ [16,17,18], there is a possibility for its interaction with GAGs to also be influenced by this metal ion, but this is yet to be examined experimentally.

In this study, we investigated the effects of HRG, fibrinogen, and fibronectin on the heparin-mediated neutralisation of factor Xa and thrombin activities. More specifically, we explored the effects of these proteins on heparins of different chain lengths and examined the potential for Zn^2+^ to play a role in these processes. Moreover, we also examined the influence of Zn^2+^ on fibrinogen–GAG interactions using surface plasmin resonance (SPR) spectroscopy. Understanding the effects of Zn^2+^ on these interactions may aid in optimising future antithrombotic therapies with the different forms of heparin.

## 2. Results and Discussion

### 2.1. Zn^2+^ Modulates Neutralisation of Heparins by HRG and Fibrinogen but Not Fibronectin

**Figure 1:**
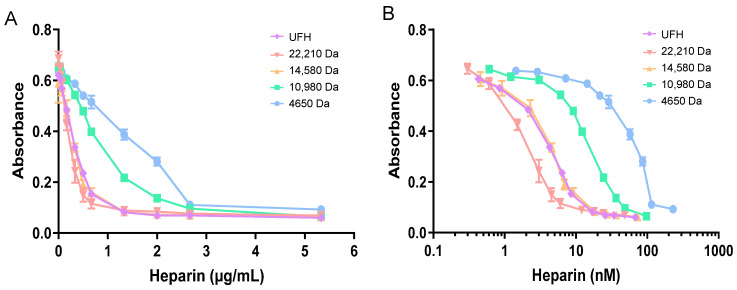
Anti-FXa activity of heparin fractions of different average molecular weights (4650–22,210 Da). (**A**) Standard curve showing effects of heparins at different concentrations on FXa activity. (**B**) Inhibition of FXa (measured as a change in absorbance) as a function of heparin concentration (MW 4650–22,210 Da). The experiments were performed in triplicate.

The inhibition of FXa by heparins of different molecular weights (anti-FXa activity) was measured in the presence of either HRG, fibrinogen, or fibronectin with and without Zn^2+^. Firstly, the anti-FXa activity of different heparin preparations (with average molecular weights ranging from 4650 Da to 22,210 Da) at different concentrations was measured (Figure 1A). For a given heparin mass concentration (reflecting both the number of saccharides and the molar concentration), increasing inhibition of FXa was observed as the chain length of the heparins increased (Figure 1B). UFH, a mixture of heparins of wide-ranging chain length with an average molecular weight of 15,700 Da, behaved in a similar manner to the purified heparin fraction closest in average molecular weight (the 14,580 Da heparin). To prepare for the next experiments, linear regression was performed to calculate the concentrations needed for each heparin to correspond to an absorbance of 0.3 AU. The resultant concentrations obtained were 5.0 nM for UFH, 2.5 nM for the 22,210 Da heparin fraction, 5.1 nM for the 14,580 Da heparin fraction, 19.0 nM for the 10,980 Da heparin fraction, and 76.0 nM for the 4650 Da heparin fraction.

**Figure 2:**
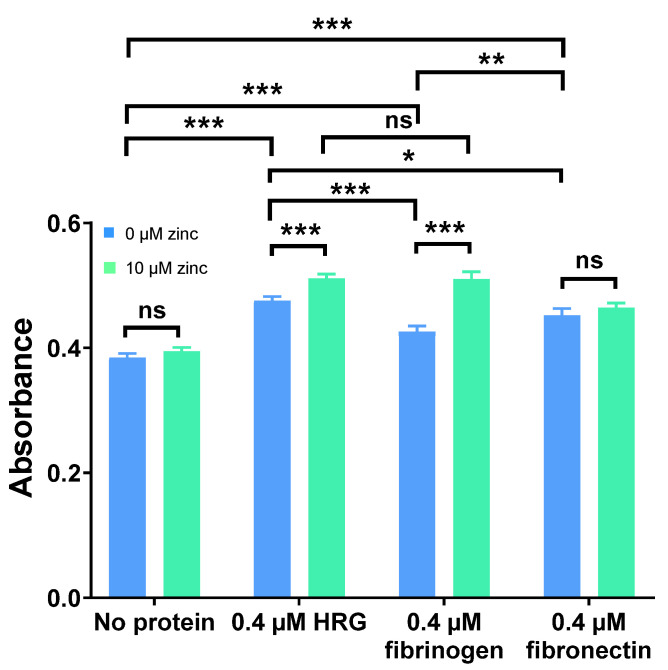
Comparison of the neutralisation of the anti-FXa activity of UFH by 0.4 µM HRG, fibrinogen, or fibronectin in the presence and absence of 10 µM Zn^2+^. The experiments were performed three times in duplicate. Addition of Zn^2+^ increased UFH neutralisation by HRG and fibrinogen but not by fibronectin. Data are represented as mean ± standard error of the mean. Statistical significance is indicated by ns (not significant) for *p* > 0.05, * for *p* < 0.05, ** for *p* < 0.01, and *** for *p* < 0.001.

Neutralisation of UFH by HRG, fibrinogen, and fibronectin and the influence of this process on inhibition of FXa was examined in the presence or absence of 10 µM Zn^2+^ (Figure 2). An increase in absorbance translated to an increase in FXa activity and thus an increased neutralisation of heparin. It was found that all three proteins neutralised anti-FXa activity of UFH, with HRG having the greatest effect, followed by fibronectin and then fibrinogen. Addition of 10 µM Zn^2+^ did not affect the anti-FXa activity of UFH in the absence of protein (so Zn^2+^ did not have any background effect) or in the presence of fibronectin. However, 10 µM Zn^2+^ increased neutralisation of UFH by HRG and fibrinogen, having a greater effect on fibrinogen than on HRG.

### 2.2. HRG and Fibrinogen Have Differential Effects on Neutralisation of Different Heparins

**Figure 3:**
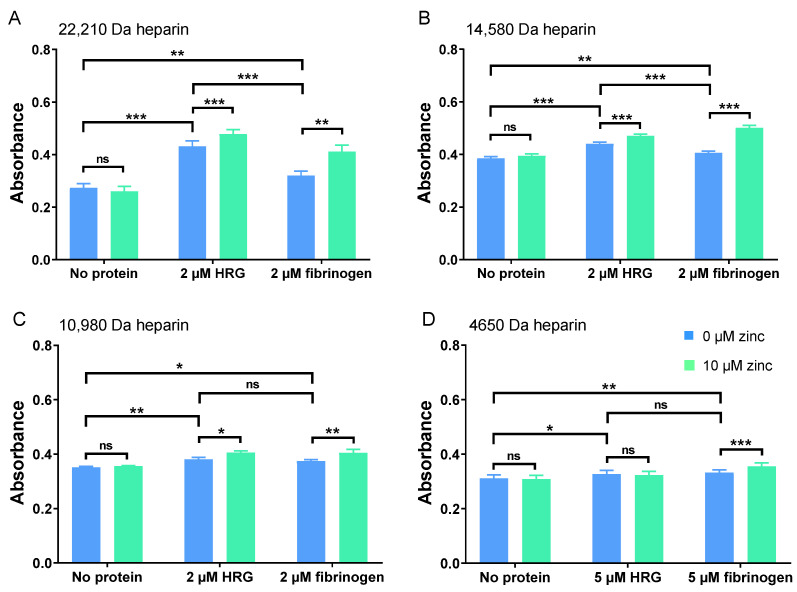
Comparison of the neutralisation of the anti-FXa activity of heparins of different molecular weight ranges by 2 µM or 5 µM HRG or fibrinogen in the presence and absence of 10 µM Zn^2+^. (**A**) Heparin averaging 22,210 Da in size (2.5 nM), (**B**) heparin averaging 14,580 Da in size (5.1 nM), (**C**) heparin averaging 10,980 Da in size (19.0 nM), and (**D**) heparin averaging 4650 Da in size (LMWH; 76.0 nM). The experiments were repeated three times in triplicate. Zn^2+^ increased the neutralisation of all heparins including LMWH by fibrinogen, but it only increased the neutralisation of longer-chain heparins by HRG. Data are represented as mean ± standard error of the mean. Statistical significance is indicated by ns (not significant) for *p* > 0.05, * for *p* < 0.05, ** for *p* < 0.01, and *** for *p* < 0.001.

The ability of HRG and fibrinogen to neutralise heparins of different molecular weights (and thus their impact on its anti-FXa activity) in the presence and absence of Zn^2+^ was then assessed (Figure 3). Partial neutralisation of 22,210 Da, 14,580 Da. and 10,980 Da heparin fractions by 0.4 µM HRG and 0.4 µM fibrinogen was observed, with HRG having a greater effect (Figure 3A–C). To examine whether a higher concentration of HRG and fibrinogen could exert an effect on this LMWH, the assay was repeated with the concentration of each protein increased to 1 µM and the heparin concentration reduced by half (down to 38.0 nM, Figure 3D). Under these conditions, some degree of neutralisation was seen with HRG and fibrinogen sufficient to see a significant change in anti-FXa activity. The smaller degree of LMWH neutralisation, occasioned by the presence of either protein, was due in part to the different heparin concentrations that had to be used in the assay to obtain adequate absorbance. However, heparin neutralisation by HRG is known to be less efficient when the molecular weight of the heparin is reduced, and this may also be the case with fibrinogen [19].

We then assessed the effect of an addition of 10 µM Zn^2+^ to the neutralisation of these heparin fractions of differing chain lengths. Zn^2+^ alone (in the absence of HRG or fibrinogen) did not significantly affect the anti-FXa activity of the heparins and so had no direct effect. Importantly, Zn^2+^ increased neutralisation by fibrinogen of heparin preparations of all molecular weight ranges, but with HRG, Zn^2+^ only increased neutralisation of the heparins with average molecular weights of 22,210 Da, 14,580 Da. and 10,980 Da (and not with the LMWH; 4650 Da). In addition, Zn^2+^ more greatly enhanced neutralisation with fibrinogen than with HRG.

### 2.3. Zn^2+^ Impacts UFH Neutralisation by HRG and Fibrinogen, but Not Fibronectin, to Enhance Thrombin Activity

**Figure 4:**
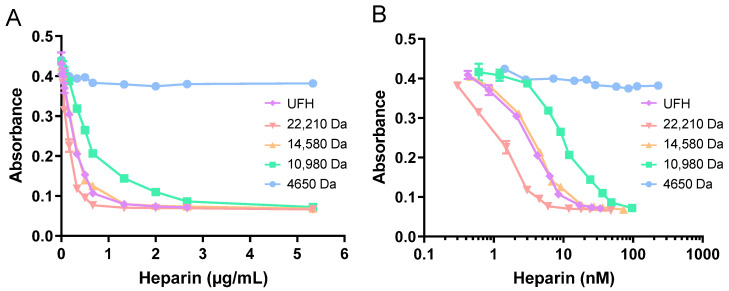
Measurement of the anti-thrombin activity of heparin fractions of different average molecular weights (4650–22,210 Da). (**A**) Standard curve showing effects of heparins at different concentrations on thrombin activity. (**B**) Inhibition of thrombin (measured as an increase in absorbance) as a function of heparin molar concentration (MW 4650–22,210 Da). The experiments were performed in duplicate. The LMWH did not influence thrombin activity.

The anti-thrombin activity of heparins of different molecular weights was compared. First, the anti-FXa activity of different heparins as a function of their concentrations was measured (Figure 4A). LMWHs (4650 Da) did not inhibit thrombin, presumably as they are too short to possess the full AT–thrombin binding site [1]. Higher-molecular-weight heparins (ranging from 10,980–22,210 Da average size) were all able to inhibit thrombin, with the degree of inhibition at each respective heparin concentration increasing with the molecular weight of the heparins. The anti-thrombin activity of UFH was then measured to compare the degree to which HRG, fibrinogen, and fibronectin neutralise heparins and to assess the influence of Zn^2+^ on neutralisation (Figure 4B). In these assays, the presence of thrombin means that the fibrinogen is transformed into fibrin. However, this does not result in a decrease in heparin neutralisation, as fibrin has been shown to form a complex with heparin and thrombin, resulting in the protection of thrombin from inactivation by AT [20], and the formation of the fibrin–heparin–thrombin complex is also enhanced by Zn^2+^ [21].

**Figure 5:**
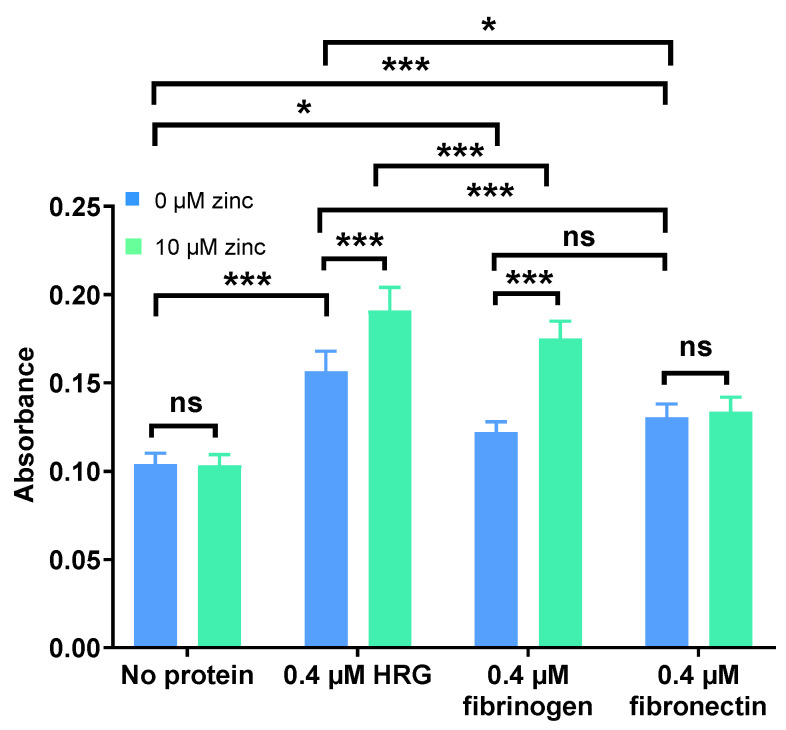
Comparison of the neutralisation of the anti-thrombin activity of UFH by 0.4 µM HRG, fibrinogen, or fibronectin in the presence and absence of 10 µM Zn^2+^. The experiments were performed in six separate replicates. HRG, fibrinogen, and fibronectin all neutralise the inhibition of thrombin by UFH, but only HRG and fibrinogen neutralisation is increased by addition of Zn^2+^. Data are represented as mean ± standard error of the mean. Statistical significance is indicated by ns (not significant) for *p* > 0.05, * for *p* < 0.05, and *** for *p* < 0.001.

HRG, fibrinogen, and fibronectin (0.4 µM) all reversed thrombin inhibition by UFH to some degree, with HRG having the greatest effect and fibrinogen and fibronectin having a smaller relative effect (Figure 5). Addition of 10 µM Zn^2+^ did not affect heparin activity in the absence of neutralising proteins. Zn^2+^ increased UFH neutralisation by HRG and fibrinogen, with the effect on fibrinogen being more pronounced. Neutralisation of UFH by fibronectin was not affected by the addition of Zn^2+^. These results show that Zn^2+^ has similar effect on the neutralisation of thrombin and FXa inhibition by UFH. As for LMWHs, the difference between the effect of Zn^2+^ on HRG and fibrinogen binding is not relevant to thrombin inhibition, as LMWHs do not have this ability. Thus, Zn^2+^ had no effect on the neutralisation of the anti-thrombin activity of heparin by fibronectin, whereas it can increase this neutralisation of UFH by both fibrinogen and HRG.

### 2.4. Zn^2+^ Enhances Fibrinogen Binding to Heparin

**Figure 6:**
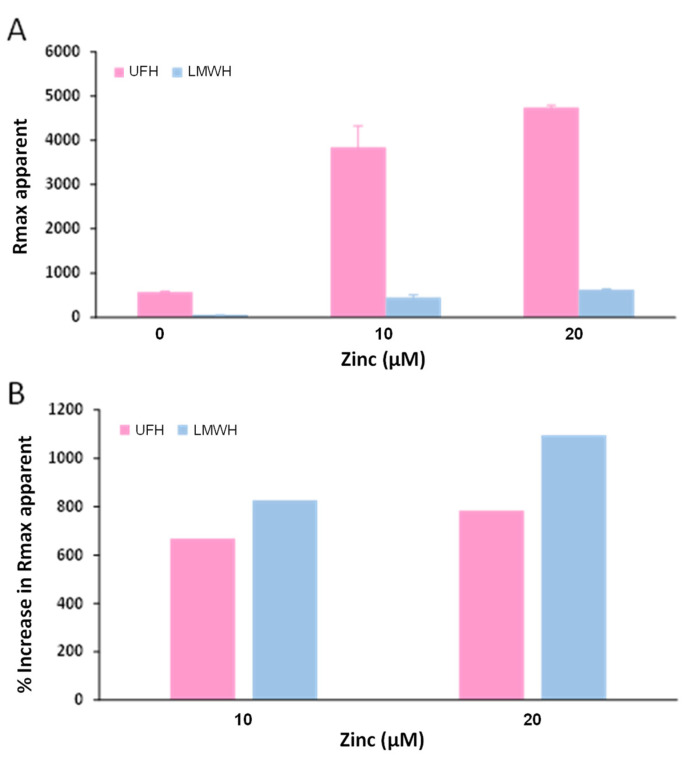
Effect of Zn^2+^ on the binding of fibrinogen to immobilised UFH and LMWH as measured by SPR. (**A**) Solutions of 250 nM fibrinogen containing 0–20 µM Zn^2+^ were injected into flow cells containing immobilised UFH or LMWH followed by a buffer wash. The RU values at maximum (Rmax apparent) obtained from the sensorgrams were corrected for binding to blank flow cells. Each experiment was repeated at least five times. Data are represented as mean ± standard error. (**B**) Average percentage increase in Rmax upon addition of 10 or 20 µM Zn^2+^.

Further experiments were then performed to better understand the effect of Zn^2+^ on the neutralisation of heparins of different sizes by fibrinogen. For this, an SPR-based approach was used to assess whether Zn^2+^ increased the affinity of fibrinogen for heparins of different molecular weights. This technique was previously used by Fredenburgh et al. to study fibrinogen binding to heparin [12]. Fibrinogen was first immobilised (up to 1002 RU) on a CM5 chip (Biacore, Uppsala, Sweden) by amination, and heparin (up to 10 µM) was injected over the flow cell in the presence of 0 or 20 µM Zn^2+^. However, only limited heparin binding to fibrinogen could be observed, and no increase in heparin binding could be seen with addition of Zn^2+^. This is probably because an amination site is present in the E domain of fibrinogen and may interfere with heparin binding nearby to the E domain or αC regions [22]. Therefore, UFH and a LMWH of 4850 Da average size were biotinylated and immobilised on a streptavidin chip. Fibrinogen samples (250 nM) containing 0, 10, or 20 µM Zn^2+^ were injected over the flow cells for 700 s. At the end of the injection, the flow cells were washed, and the RU was immediately measured. The apparent Rmax was plotted against Zn^2+^ concentration (Figure 6). The binding of both UFH and the LMWH to fibrinogen increased with addition of Zn^2+^ in a concentration-dependent manner up to 20 µM Zn^2+^.

To summarise, HRG, fibrinogen, and fibronectin were found to neutralise the ability of heparins to inhibit thrombin and FXa when bound to AT. Due to the structural and biochemical similarities between heparin and heparan sulphate, those proteins are also likely able to neutralise the anticoagulant activity of the heparan sulphate on the endothelial surface layer, thus negating the natural anticoagulant properties of the endothelium. In addition, platelets are activated during coagulation and release Zn^2+^. As described before, this increase in the local plasma Zn^2+^ concentration is likely sufficient to increase the affinity of HRG for heparins (but not for LMWHs) [8]. The results presented here show that Zn^2+^ also increases the binding rate (and so possibly the affinity) of fibrinogen for heparins, including LMWHs. Furthermore, the effect of Zn^2+^ on heparin binding also leads to an increased neutralisation of heparin (excluding LMWHs for HRG) and thus to an increase in FXa and thrombin activity (pro-coagulant activity).

Interestingly, another heparin-neutralising protein, platelet factor 4 (PF4), is known to complex with proteoglycans on the surface of platelets [23]. It has been known for some time that Zn^2+^ can be used to precipitate PF4 and aid in its extraction and purification from platelet preparations and that pre-treatment of zinc-precipitated PF4 with EDTA abolishes its para-coagulating properties, which may also suggest a role for Zn^2+^ in regulating its activities [24].

The anticoagulant neutralising properties of HRG, fibrinogen, and fibronectin are of particular relevance, as those three proteins are released by activated platelets alongside Zn^2+^ [3]. This neutralisation at the endothelium helps to enable coagulation to take place, an effect that is enhanced by the local increase in Zn^2+^ concentration (for HRG and fibrinogen). This action is desirable in normal circumstances when coagulation is required. However, if Zn^2+^ concentration is dysregulated, as is the case in the presence of pathologically elevated plasma FFA concentrations (which disrupt the albumin–Zn^2+^ buffering via an allosteric mechanism) [25], this may negate an important endothelial protection pathway that safeguards against undesired coagulation. In addition to the effect of Zn^2+^ on fibrin clotting, this provides another mechanism by which elevated Zn^2+^ concentrations can result in an increase in thrombotic risk in diseases associated with elevated plasma FFA concentrations. Finally, it is important to note that fibrinogen is better able to neutralise LMWHs than HRG, which is further enhanced by Zn^2+^. As the plasma concentration of fibrinogen is ten times greater than HRG (with an average of around 12–24 µM compared to 1.3–2.0 µM) [26,27,28], this makes fibrinogen the most relevant protein of the two for LMWH neutralisation, even if HRG is an important neutraliser of HMWHs. LMWHs are more widely prescribed than UFH, as their smaller size leads to fewer interactions with other molecules in the plasma and thus provides more stable inhibition of coagulation with a longer effect and without the need for constant monitoring [1]. However, the results presented here show that some interaction/neutralisation still occurs, and this may be important clinically.

To optimise LMWH therapies, future clinical work should consider optimisation of these therapies according to fibrinogen levels, which our data show can modulate the antithrombotic efficacy of these agents. Moreover, given the Zn^2+^ interactions with both HRG and fibrinogen, further refinement of these therapies needs to consider plasma zinc levels and even FFA levels (that can displace Zn^2+^ from albumin) [26]. This will allow for personalised therapies, thus maximising the antithrombotic effects of LMWH while limiting bleeding complications. Admittedly, UFH is also affected by fibrinogen, HRG, and fibronectin as well as zinc levels, but such a treatment is usually closely monitored using appropriate coagulation assays, and therefore, measuring levels of these proteins or assessing zinc levels is perhaps less relevant. In conclusion, our results show that an increase in Zn^2+^ concentration can increase anticoagulant GAG neutralisation by HRG and fibrinogen, thus influencing coagulation in plasma.

## 3. Materials and Methods

### 3.1. Materials

UFH with an average molecular weight of 15,700 Da (size range of 5000 to 30,000 Da) and heparin preparations of more narrow size ranges (purified by fractionation) with average molecular weights of 22,210 Da; 149,580 Da; 10,980 Da; 4850 Da; and 4650 Da were purchased from Iduron (Cheshire, UK). The heparin concentrations detailed in this study are based upon dissolution of known weights of the lyophilised heparin in appropriate volumes of buffer. Calculations were based on the average formula weight of the respective heparin preparation. Fibrinogen purified from human plasma and depleted of plasminogen was bought from Merck (Dorset, UK). and fibronectin purified from human plasma was bought from Sigma (Poole, UK). HRG was purified from human plasma using the method described below.

### 3.2. Purification of HRG from Human Plasma

HRG was purified from commercially obtained human plasma (First Link Ltd., Wolverhampton, UK) using Ni^2+^-NTA affinity chromatography. The protocol employed the use of a 5 mL HisTrap column (Cytiva, Little Chalfont, UK) and an ÄKTA Pure FPLC system (Cytiva). First, plasma was centrifuged (4000× *g* for 30 min at 4 °C); imidazole was added (to a final concentration 5 mM) before filtering through a 0.45 µm filter (Sartorius, Epsom, UK). The column was equilibrated with 10 column volumes of the equilibration buffer (5 mM sodium phosphate, 50 mM NaCl, and 5 mM imidazole, pH 7.4). After loading the column with the sample (50 mL), the column was washed with 10 column volumes of a 20 mM sodium phosphate, 50 mM NaCl, and 20 mM imidazole pH 7.4 buffer. Elution was performed with 20%, 40%, 60%, 80%, and 100% of elution buffer (500 mM imidazole, 50 mM NaCl, and 5 mM sodium phosphate, pH 7.4), with each elution step carried out with 10 column volumes of elution buffer/equilibration buffer mix. The fractions containing HRG were concentrated to 2.5 mL using a Vivaspin centrifugal concentrator with 50 kDa molecular weight cut-off (Sartorius). The protein was then further purified by size-exclusion chromatography using a HiLoad Superdex 26/600 200 pg column (Cytiva). The column was prepared for purification with 1 column volume of 100 mM NaOH at 2.6 mL/min, 2 column volumes of water, and 1.5 column volume of a 10 mM Tris, and 150 mM NaCl pH 7.4 buffer. After loading the column with 2.5 mL of the concentrated protein mixture, elution was performed at 0.6 mL/min with 1.5 column volume of 10 mM Tris, and 150 mM NaCl at pH 7.4.

### 3.3. Anti-FXa and Thrombin Assays

Heparins bind anti-thrombin (AT) in a manner that enhances its inhibition of thrombin and FXa [1,2]. Anti-Xa heparin and anti-IIa heparin kits (both from Iduron) were used to assess the degree to which FXa or thrombin activities, respectively, are reduced by heparin (thus measuring the anti-FXa or anti-thrombin activity of heparin) and to determine how the presence of different proteins (fibrinogen, HRG, and fibronectin) and Zn^2+^ influence heparin activity. All samples and reagents were pre-incubated at 37 °C. AT was added to heparin, and they were incubated 2 min at 37 °C to form an AT–heparin complex. For anti-FXa assays, a known excess of FXa was added and the solution incubated 2 min at 37 °C. FXa bound to AT–heparin, forming an inactive complex and leaving some residual FXa. A colourless FXa substrate (Cbo-D-Arg-Gly-Arg-pNA∙2HCl) was added, and they were incubated 2 min at 37 °C. The substrate reacted with the residual FXa to form the chromophore molecule pNA (detectable at 405 nm) and a peptide. For anti-thrombin assays, a known excess of thrombin was added, and the solution was incubated 2 min at 37 °C. Thrombin bound to AT–heparin, forming an inactive complex with some residual thrombin. A colourless thrombin substrate (H-D-Phe-Pip-Arg-pNA∙2HCl) was added, and the solution was incubated for 2 min at 37 °C. The substrate reacted with the residual thrombin to form the chromophore molecule pNA (detectable at 405 nm) and a peptide. The assays were carried out in triplicate (for anti-FXA assays) or in duplicates (for anti-thrombin assays) in a 50 mM Tris and 150 mM NaCl pH 7.4 buffer (tris buffer saline, TBS) containing different concentrations of heparins (0–10 µg/mL for anti-FXa assays; 0–5 µg/mL for anti-thrombin assays) ranging in average size (4650–22,210 Da). To each well in a 96-well plate, 50 µL of each heparin sample was added and incubated 2 min at 37 °C. Following this, three mixtures were added in succession. For anti-FXA assays, 50 µL of AT (0.5 IU/mL in TBS), 50 µL of FXa (2.5 µg/mL in TBS), and 50 µL of FXa substrate (0.5 mg/mL in H_2_O) were added. For anti-thrombin assays, 50 µL of AT (0.25 IU/mL in TBS), 50 µL of thrombin (4 IU/mL in TBS), and 50 µL of thrombin substrate (0.625 mg/mL in purified H_2_O) were added. After each step, the wells were mixed four times, and the plate was incubated for 2 min at 37 °C. The reaction was stopped by addition of 50 µL of 20% (*v*/*v*) acetic acid. The final solutions were mixed four times, and the absorbance was read immediately at 405 nm. The absorbance was plotted against the heparin concentrations. To obtain maximum sensitivity, the heparin concentration needed to obtain an absorbance of 0.3 absorbance units (AU) in the anti-FXa assays was calculated for each heparin. Heparins at these corresponding concentrations were then mixed with either 0.4 or 1 µM HRG, fibrinogen, or fibronectin and 0 or 10 µM Zn^2+^ diluted in TBS. Assays were performed with those samples in triplicate with each individual assay repeated at least twice for the anti-FXa assays and in six replicates for the anti-thrombin assays. Differences between the samples were analysed using two-way ANOVA followed by Sidak’s multiple comparisons tests. Data are shown as the mean ± the standard error of the mean (SEM). The significance threshold was set at *p* ≤ 0.05. Statistical analyses were performed, and graphs generated using Prism 7.0 (GraphPad, San Diego, CA, USA).

### 3.4. Surface Plasmon Resonance (SPR)

SPR experiments were performed following a previously described protocol [12]. UFH and a LMWH (with an average molecular weight of 4850 Da) were biotinylated. It was previously demonstrated that heparins are functional after biotin conjugation [29]. For biotinylation, 14 mg/mL heparin diluted in H_2_O was mixed with (+)-biotinamidohexanoic acid hydrazide (13 mg/mL diluted in 0.1 M sodium acetate pH 5.5 buffer) and incubated for 2.5 h in a shaker at 23 °C. The heparins were then extensively dialysed over a period of 3 days in 5 L buffer of 50 mM HEPES, 150 mM NaCl, and 0.2% Tween 20 at pH 7.4 (buffer was replaced each day). Biotinylated heparin was immobilised on an SA chip (with streptavidin bound at the surface of the flow cells; Biacore, Cytiva) following the manufacturer instructions and using a Biacore T200 instrument (Cytiva). The immobilisation target was set at 640 resonance units (RU), and the buffer used was 50 mM HEPES, 150 mM NaCl, and 0.2% Tween 20 at pH 7.4. Aliquots of 250 nM fibrinogen were dialysed overnight at room temperature against 5 L of the same buffer containing 0–20 µM Zn^2+^. They were then injected for 700 s at 30 µL/min over the flow cells in five repeats. The flow cells were regenerated with a solution containing 0.5% SDS and 1 mM EDTA. The RU of reference flow cells was subtracted from the RU of the flow cells where heparin was bound. The RU values at stability were then calculated and plotted against Zn^2+^ concentrations. Graphs were generated using Prism 7.0.

## Data Availability

The data generated in the present study may be requested from the corresponding author.
